# Transcranial alternating current stimulation combined with sound stimulation improves the cognitive function of patients with Alzheimer's disease: A case report and literature review

**DOI:** 10.3389/fneur.2022.962684

**Published:** 2022-09-23

**Authors:** Yang Liu, Can Tang, Kailun Wei, Di Liu, Keke Tang, Meilian Chen, Xuewei Xia, Zhiqi Mao

**Affiliations:** ^1^Department of Neurosurgery, Affiliated Hospital of Guilin Medical University, Guilin, China; ^2^Guangzhou Kangzhi Digital Technology Co., Ltd., Guangzhou, China; ^3^Department of Neurosurgery, Chinese PLA General Hospital, Beijing, China

**Keywords:** Alzheimer's disease, cognition, transcranial alternating current stimulation, gamma rhythm, sound

## Abstract

Transcranial alternating current stimulation (tACS) is a relatively new non-invasive brain electrical stimulation method for the treatment of patients with Alzheimer's disease (AD), but it has poor offline effects. Therefore, we applied a new combined stimulation method to observe the offline effect on the cognitive function of patients with AD. Here, we describe the clinical results of a case in which tACS combined with sound stimulation was applied to treat moderate AD. The patient was a 73-year-old woman with a 2-year history of persistent cognitive deterioration despite the administration of Aricept and Sodium Oligomannate. Therefore, the patient received tACS combined with sound stimulation. Her cognitive scale scores improved after 15 sessions and continued to improve at 4 months of follow-up. Although the current report may provide a new alternative therapy for patients with AD, more clinical data are needed to support its efficacy.

**Trial registration:**

Clinicaltrials.gov, NCT05251649.

## Introduction

Alzheimer's disease (AD) is a severe neurodegenerative disease that has been among the top 10 causes of death worldwide and has caused a substantial economic burden on families (1). The characteristic performance of patients with AD is a gradual deterioration of cognition (2). However, existing drugs and some treatments have not achieved good results in improving cognition.

A relatively new non-invasive method for brain electrical stimulation is transcranial alternating current stimulation (tACS). It affects the manipulation and entrainment of intrinsic oscillations of the brain through the sinusoidal waveform current and regulates the oscillatory activity of cortical regions (3, 4). tACS can interact with ongoing neuronal activity during cognitive processes, leading to changes in cognitive function (4, 5). tACS regulates higher-order cognitive processes, including memory (4, 6). It can be applied to the phase of coding and identification in learning and memory (7, 8). In addition, tACS is a safe form of stimulation and may have mild and transient side effects without serious adverse events (9). Therefore, tACS can be used to improve the cognition of patients with AD. Nevertheless, so far, tACS has only a temporary effect on cognitive improvement in patients with AD. However, the cognition of a patient with AD' gradually deteriorates as the disease worsens. Therefore, how to extend the offline effects of the treatment has become an urgent problem that needs to be solved.

## Case report

### Participant

Ms. Hu, aged 73, had a BMI of 24.2, normal blood pressure, primary school education, was a non-smoker and alcoholic (250 ml/day), and had undergone heart stent surgery. And she has no family history of AD. Diagnosed with AD for 2 years, taking Aricept (Donepezil Hydrochloride tablets) for 2 years and taking Sodium Oligomannate capsules for 1 year and still continuing, cognitive deterioration is still progressing. Moreover, the patient complained of a continuous rather than a sudden or volatile decline in memory to our hospital. Impaired ability to acquire and remember new information, as well as reason and process complex tasks, poor judgment, changes in personality or behavior, and occasional anxiety disrupted her daily activities. We assessed this patient on multiple psychometric scales at baseline, including: Alzheimer's Disease Assessment Scale-Cognitive subscale (ADAS-Cog) (10); Montreal Cognitive Assessment (MoCA) (11); Mini-Mental State Examination (MMSE) (12); Auditory Verbal Learning Test (AVLT); Clinical Dementia Rating (CDR) and Beck Anxiety Inventory (BAI). The scores are shown in Table 1.

**Table 1:** Raw score of all scales.

**Scales**	**Sub-items**	**Baseline**	**Post-intervention**	**Follow-up**
ADAS-Cog		17	14	10
	Word recall tasks	1	1	1
	Object and finger naming	1	1	1
	Execution of commands	0	0	0
	Structural exercises	3	3	1
	Imagery exercises	0	0	0
	Orienting power	5	4	5
	Word recognition tests	6	5	2
MoCA		12	14	16
MMSE		15	16	18
AVLT
	Immediate recall	7	8	8
	Delayed recall (5 min/20 min)	0/0	1/0	2/2
	Recognition	10	16	16
BAI		4	6	4

### Transcranial alternating current stimulation

The alternating current was non-invasively delivered using a transcranial electrical current stimulator (XPNS208-B, Suzhou Hypnos MD Co. Ltd., China). She received tACS with gamma frequency (40 Hz) and a peak-to-peak amplitude of 1.5 mA 15 times with 20-min sessions across 3 weeks (21 days). Two electrodes (4 × 6 cm) were placed in the dorsolateral prefrontal cortex and in the contralateral supraorbital area.

### Sound stimulation

**Figure 1:**
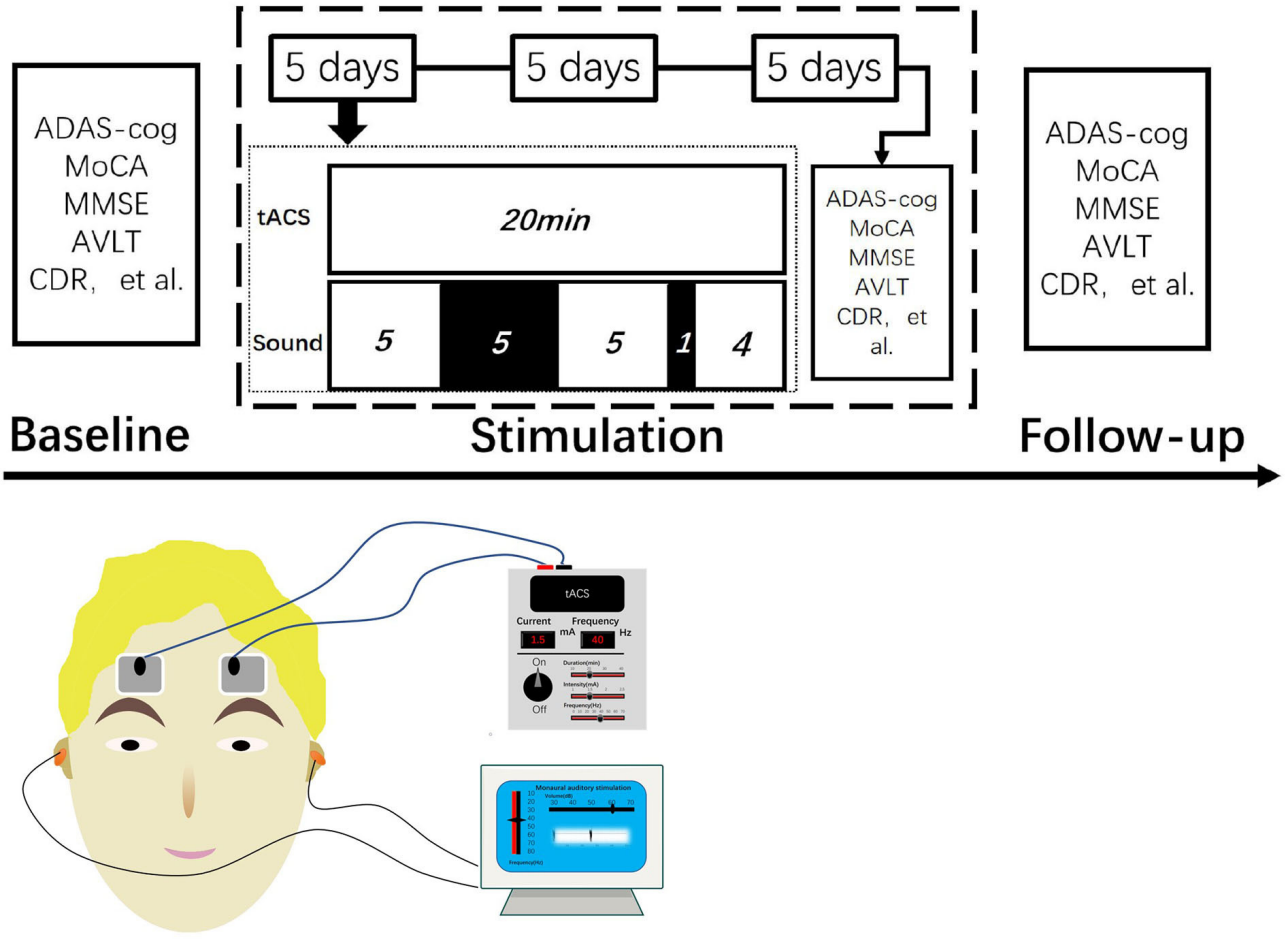
Test process and stimulus mode.

By placing two sponge earbuds in the ear and setting the sound stimulator to 40 Hz, 60 dB, and pure tone, the patient was able to hear clearly. The beginning of tACS coincided with the simultaneous activation of sound stimulation. The time of sound stimulation was set for 5-min stimulation, 5-min rest, 5-min stimulation, 1-min rest, and continued stimulation until tACS ended while turning off sound stimulation. tACS and sound stimulation started and ended at the same time (Figure 1).

### Assessments

We measured the patient on several neuropsychological assessment scales. They included CDR, ADAS-Cog, MoCA, MMSE, AVLT, and BAI. All assessments were measured three times: baseline, after the intervention (the end of the treatment for 3 weeks), and follow-up (4 months after the intervention). As the main purpose of this treatment is to evaluate the cognitive effect of this double stimulation method on patients with AD, the main outcome is the change in the ADAS-Cog score, and the other scales are the secondary outcomes. After each treatment, this patient is questioned regarding any adverse reactions such as headaches, itchy skin, dizziness, flickering lights, tinnitus, fatigue, drowsiness, and acute mood changes.

### Outcome

**Figure 2:**
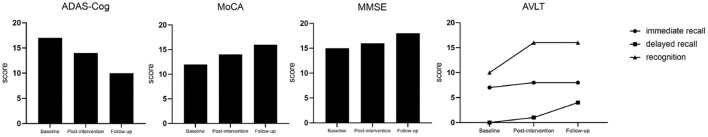
The changing trend of several cognitive scale scores. ADAS-Cog, Alzheimer's Disease Assessment Scale-Cognitive Scale; MoCA, Montreal Cognitive Assessment; MMSE, Mini-Mental State Examination; AVLT, Auditory Verbal Learning Test; CDR, Clinical Dementia Rating.

After 15 days of treatment, the patient was re-evaluated with the scales. It was measured that the ADAS-Cog score dropped by three points, the MoCA score increased by two points, the MMSE score increased by one point, the AVLT score improved by one point for immediate recall, by one point for delayed recall (5 min), and by up to 6 points for recognition. We found that the ADAS-Cog score was decreased by seven points compared with the baseline in a 4-month follow-up, and the MoCA and MMSE scores both improved by two points compared with scores after the intervention, and the delayed recall of AVLT (5 and 20 min) was improved by two points compared with baseline (Figure 2, Table 1). Although the patient had self-reported anxiety symptoms, the BAI scale did not reveal anxiety.

### Literature review

We searched the iterature from 2016.01 to 2022.05 in the Web of Science and PubMed databases. Based on Preferred Reporting Items for Systematic Evaluation and Meta-Analysis (PRISMA) guidelines, we performed a systematic evaluation of the available literature related to γ-tACS for the treatment of AD. A literature search was performed using the following terms in the search query. An initial search yielded 30 articles, and two additional studies were obtained in the primary literature reference. A screening for relevance was also performed. After excluding duplicates, reviews, animal experiments, and reports that did not meet the inclusion criteria, three reviewers screened the titles and abstracts of the papers to limit errors. Full-text articles were reviewed, and articles were manually reference checked to show additional references that were missed in the original search. Finally, we found seven articles with data relevant to our research. Considering the limited quantities of clinical trials where γ-tACS was applied to patients with AD, all case reports and small series were eligible. Cases were reported if they met the inclusion criteria. The extracted data included the following parameters: (1) references; (2) participants and design; (3) grouping; (4) electrode position; (5) frequency; (6) intensity; (7) duration; (8) follow-up; (9) assessment; and (10) outcome. Table 2 summarizes the seven studies included.

**Table 2:** Clinical trials of transcranial alternating current stimulation (tACS) for patients with Alzheimer's disease (AD) and stimulation parameters.

**Reference**	**Participants and design**	**Grouping**	**Electrode position**	**Frequency**	**Intensity**	**Duration**	**Follow-up**	**Assessment**	**Outcome**
Dhaynaut et al. (13)	4 AD	γ-tACS	Bitemporal lobes	40 Hz	2 mA	1 h, 4 w	-	ADAS-Cog, MMSE	No significant changes in overall cognition tests
Sprugnoli et al. (14)	15 AD	2 w/unilateral 2 w/bilateral 4 w/bilateral	Temporal lobes	40 Hz	4 mA	1 h, 2 w/4 w	-	ADAS-Cog, MMSE, MoCA CS 21, CFT (15)	No significant changes in overall cognition tests
Kim et al. (16)	20 MCI repeated-measures	γ-tACS; tDCS; sham-tACS	Prefrontal cortex	40 Hz	2 mA	30 min	-	Stroop test, TMT	The cognitive benefits of tACS superior tDCS
Benussi et al. (17)	20 MCI-AD cross-over study	γ-tACS; sham-tACS	Pz; right deltoid muscle	40 Hz	3 mA	1 h	-	RAVL FNAT	Memory improved
Bréchet et al. (18)	2 ADRD	γ-tACS	LAG	40 Hz	2 mA	20 min, 14 w	-	MoCA	Memory improved
Zhou et al. (19)	50 AD randomized, controlled	γ-tACS; sham-tACS	Bilateral temporal lobes	40 Hz	2 mA	20 min, 6 w	12 w	ADAS-cog, MMSE	Cognitive improvement but not sustained
Kehler et al. (20)	17 dementia	(γ-tACS/sham-tACS) + brain exercise	LDLPFC; contralateral supraorbital area	40 Hz	1.5 mA	30 min, 4 w	1 m	WMS-IV	Memory improved at post-intervention and follow-up.

ADAS-Cog, Alzheimer's Disease Cognitive Component Assessment; MMSE, Mini-Mental State Examination; MoCA, Montreal Cognitive Assessment; CS 21, Craft Story 21; CFT, Category Fluency Task; TMT, Trail-Making Test; RAVL, Rey auditory Verbal Learning Test; FNAT, face-name associations task; WMS-IV, Wechsler Memory Scale; ADRD, Alzheimer' s disease-related dementia; Pz, an area overlying the medial parietal cortex and the precuneus; LAG, left angular gyrus; LDLPFC, left dorsolateral prefrontal cortex.

## Discussion

Although very few trials have been conducted on patients with AD, tACS has been shown to improve cognitive ability in healthy subjects (21), and its immediate effects on improved patient cognition are known based on existing trials. A single 60-min treatment with exposure to γ-tACS over Pz (an area overlying the medial parietal cortex and the precuneus) can improve memory function in patients with mild cognitive impairment due to Alzheimer's disease (MCI-AD) (17). There was a beneficial effect on cognition after intervention in two patients with Alzheimer's disease-related dementia (ADRD) for a 14-week long-term tACS (18). Patients with mild cognitive impairment (MCI) or dementia received 40 Hz tACS twice a day for 4 weeks, and their' memory improved after treatment and a 1-month follow-up (20). In a randomized controlled clinical trial of 50 patients with AD subjected to 40-Hz tACS (2 mA, 20 min) for 6 weeks, patients in the treatment group showed improvements in both the MMSE and ADAS-Cog scores after treatment. However, a downward trend toward both scale scores was found in a 12-week follow-up compared to post-treatment (19). This is the largest clinical trial to date involving tACS in patients with AD. The ADAS-Cog and MMSE scores did not improve over the application of 40-Hz tACS (2 mA, 1 h) for 4 weeks compared with pre-treatment. However, after treatment, gamma spectral power on the electroencephalogram (EEG) increased, and the phosphorylated Tau showed a significant decrease following tACS. Most of the current trials reported the short-term efficacy of tACS for AD, but only two trials have patient follow-up data. However, cognitive improvements in patients with AD did not last in a follow-up.

However, we are more concerned about the offline effects of cognitive improvement in patients with AD. To our knowledge, this is the first treatment that combines electrical stimulation and sound stimulation to improve the cognition of patients with AD. The addition of 40-Hz sound stimulation to 40-Hz tACS alone distinguishes our trial from previous stimulation modalities reported in the literature. Our comparison of this treatment modality with previous studies showed surprising results in a follow-up despite the fact that there were no significant differences in cognitive scale improvements after treatment. Surprisingly, the effect was maintained 4 months after treatment. This may be due to electrical stimulation of the cerebral cortex, which causes the current to spread through the skull and activate a broad brain volume, activating multiple interconnected neural regions (22, 23). The effect of tACS can reach the deep structure of the brain (24). Sound stimulation can activate local neuronal populations that overlap with the extensive activation induced by surface electrical stimulation. Therefore, this coordinated activation of these two modalities can lead to enhanced modulation or paired plasticity within localized overlapping areas (25, 26). tACS increases auditory stimulus-evoked power and phase locking (27). Both stimuli were performed simultaneously, resulting in better treatment outcomes. As demonstrated by Conlon et al., a combination of electrical stimulation and sound stimulation effectively modified tinnitus, and the effect lasted for 12 months after treatment. Also, only two kinds of stimulation simultaneously produce the best effect (28).

We enrolled this patient with AD according to the NIA-AA label (29). Our findings are consistent with previous findings that tACS can improve patient cognition. This patient was assessed on the cognitive scales after treatment and showed an improvement compared to baseline. However, cognitive scales scores were better in a 4-month follow-up than in post-treatment. Patients with moderate AD can reduce the total ADAS-Cog score by 6–10 points per year without treatment. A 4-point improvement is usually used to determine the effectiveness of clinical anti-dementia therapy (10). In this treatment, the patient's ADAS-Cog can effectively reduce the score by seven points from baseline to follow-up, indicating that the tACS combined with sound stimulation improves the cognition of a patient with AD significantly, and there is an offline effect. Through the sub-scale analysis and summary of each scale, we found better improvements in recognition (ADAS-Cog word recognition test, AVLT recognition), visual perception, and attention. Recognition is an essential process of memory. Memory is the ability of humans to encode, store, and extract information, which plays an important role in daily life. Significantly, patients with AD have impaired memory that interferes with daily life. The AVLT scale, a measurement tool used to check episodic memory, also showed improved delayed recall score compared to the baseline after intervention and follow-up. In addition, the patient did not experience any adverse effects after each treatment. The patient and her family actively cooperated with each treatment. Although the patient said there was no significant change in cognition before and after the treatment, the patient's mood improved after the treatment and the tension in answering questions was relieved according to the statements by the patient's family members and the observation of a psychological evaluator.

The advantage of using tACS in this experiment is that its current can oscillate at a specific frequency and interact with the intrinsic oscillation of the brain. Selecting the stimulation frequency is significant for tACS to improve the cognition of patients with AD. We summarized the literature and found that most of the published trials for the treatment of patients with AD applied 40 Hz as the stimulation frequency of tACS. As gamma oscillations are closely related to cognitive function, they are impaired in multiple AD models (30). Approximately 40 Hz is the stimulation frequency used in this experiment because, in previous studies, it has been found that 40-Hz light flicker or 40-Hz tone flickering is applied to an animal or 40-Hz tACS is applied to a patient with AD can improve cognitive function and decrease amyloid and phosphorylated tau levels (13, 31, 32). The role of gamma frequency (35–48 Hz) in advanced cognitive processes (including attention, learning, and memory) has been studied (33, 34).

Gamma oscillations may play a more fundamental or universal role in the advanced cognitive function of the brain (35). During sensory processing or memory encoding, the power of gamma oscillation activity increases. A reduction in gamma oscillations is observed in the brain of patients with AD (36, 37). Therefore, the adjustment of the gamma band in patients with AD may help improve cognitive function (38).

Previously, in a mouse model of AD, the restoration of appropriate levels of interneuron-specific sodium channel proteins and parvalbumin (PV) cell-predominant sodium channel proteins was found to improve gamma oscillations and cognitive functions (e.g., spatial learning and memory retention) (39–41). The interneuron network is most sensitive to 40-Hz stimulation (42). After 1 h of light stimulation at 40 Hz, amyloid-β protein content in the visual cortex of mice can be reduced. Interestingly, 1 h of stimulation repeated for a week reduced not only amyloid levels in older mice but also neurotoxic plaque load as well as tau protein accumulation in the frontotemporal dementia model and increased the activity of brain immune cells (i.e., microglia) (43). Memory performance in mouse models of AD can be improved by reducing amyloid protein loads and the spread of tau phosphorylation in the auditory cortex and the hippocampus using 40-Hz auditory stimulation at 1 h for 7 days (32). Sound stimulation can affect neuroelectric activity (44). In addition, when combining auditory and visual gamma stimulation, amyloid-β clearance was increased not only in the primary sensory cortex and the hippocampus but also in the medial prefrontal cortex and even throughout the neocortex (32). The most important complex pathophysiology of AD is the accumulation of amyloid-β and tau proteins. It can be seen that this method of combining multiple stimuli can better improve cognitive function.

Sensory stimulation with gamma frequency can improve synaptic function, enhance nerve protection factors, and reduce neuronal damage (45). Furthermore, this effect is well-expressed at a frequency of 40 Hz. Thus, the regulation of neuronal gamma activity using 40-Hz stimulation represents a novel non-invasive, non-pharmacological approach to patients with AD (38). The patient underwent tACS combined with sound stimulation treatments 15 times. During each treatment and follow-up, adverse reactions were evaluated. The patient had no headaches, itching, tinnitus, and other adverse reactions during the whole treatment period, and she was willing to receive treatment and actively cooperate with us during a follow-up. Therefore, safety, tolerability, and patient compliance with this treatment method can be confirmed. This preliminary study provides evidence to support the positive effects of tACS combined with sound stimulation on the cognition of patients with AD. Therefore, this combination therapy is expected to become a new method to improve cognition in patients with AD in the future. This case report also provides a new idea for treating patients with AD in the future. This trial paradigm has a good effect on improving the cognition of this patient with AD, but, in terms of stimulation frequency and duration, it continues to improve. Finding the best stimulation parameters is a question that we have explored and researched in the electrical stimulation therapy.

Being considered a novel treatment, it is unclear which mechanism improves cognition and has a sustained offline effect on patients with AD. Because only one patient with moderate AD received such treatment in this study, no general conclusions can be drawn. In addition, unfortunately, in order to study the effects of such stimulation modalities on brain network connectivity, this patient has undergone functional magnetic resonance imaging (MRI) measurements post- and pre-treatment, but studies of brain network connectivity require the combination of functional magnetic resonance imaging (fMRI) data from multiple patients, so the effect of this treatment on brain network connectivity could not be determined here. Additional patient data are currently being collected to confirm these results. Future studies will explore the detailed mechanisms underlying the effects of tACS combined with sound stimulation in patients with mild to moderate AD. The efficacy of tACS combined with sound stimulation is found in patients with moderate AD. As the patient with AD was able to self-manage (e.g., able to feed themselves, dress themselves, go up and down stairs alone, etc.) except for impaired cognitive function, the effect of this therapy on the functional and behavioral symptoms of patients with AD was not reported in this study. Although this patient had a 4-month follow-up, we would follow her for a longer period to explore the prolonged offline effects of tACS combined with sound stimulation.

We report a novel therapy that combines tACS with sound stimulation to improve cognition in patients with AD. This method produced a significant cognitive improvement in this patient, with no adverse effects.

## Data availability statement

The original contributions presented in the study are included in the article/supplementary material, further inquiries can be directed to the corresponding author/s.

## Ethics statement

The studies involving human participants were reviewed and approved by Chinese PLA General Hospital. The patients/participants provided their written informed consent to participate in this study. Written informed consent was obtained from the individual(s) for the publication of any potentially identifiable images or data included in this article.

## Author contributions

Patients with AD were enrolled and treated by DL. Diagnosis of patients with AD was carried out by ZM. Data collection and analysis were carried out by KT and MC. The first draft of this manuscript was written by YL and KW. The experiment was designed by YL, XX, and ZM. The scale was evaluated by YL. English translation was done by CT. All authors contributed to the study conception and design, commented on previous versions of this manuscript, and read and approved the final manuscript.

## Funding

This study was supported by grants from the China Brain Project (2021ZD0200407), the National Natural Science Foundation of China (No. 81871087), the Innovative Technique Project of Chinese PLA General Hospital (XJS-202103), and the National Clinical Research Center for Geriatric Diseases (No. NCRCG-PLAGH-2018006).

## Conflict of interest

Authors DL, KT, and MC were employed by Guangzhou Kangzhi Digital Technology Co., Ltd.

The remaining authors declare that the research was conducted in the absence of any commercial or financial relationships that could be construed as a potential conflict of interest.

## Publisher's note

All claims expressed in this article are solely those of the authors and do not necessarily represent those of their affiliated organizations, or those of the publisher, the editors and the reviewers. Any product that may be evaluated in this article, or claim that may be made by its manufacturer, is not guaranteed or endorsed by the publisher.
